# A wavelet-based decomposition method for a robust extraction of pulse rate from video recordings

**DOI:** 10.7717/peerj.5859

**Published:** 2018-11-27

**Authors:** Miha Finžgar, Primož Podržaj

**Affiliations:** Faculty of Mechanical Engineering, University of Ljubljana, Ljubljana, Slovenia

**Keywords:** Remote photoplethymosgraphy, Image processing, Heart rate, Pulse rate, Signal processing, Biomedical monitoring

## Abstract

**Background:**

Remote photoplethysmography (rPPG) is a promising optical method for non-contact assessment of pulse rate (PR) from video recordings. In order to implement the method in real-time applications, it is necessary for the rPPG algorithms to be capable of eliminating as many distortions from the pulse signal as possible.

**Methods:**

In order to increase the degrees-of-freedom of the distortion elimination, the dimensionality of the RGB video signals is increased by the wavelet transform decomposition using the generalized Morse wavelet. The proposed Continuous-Wavelet-Transform-based Sub-Band rPPG method (SB-CWT) is evaluated on the 101 publicly available RGB facial video recordings and corresponding reference blood volume pulse (BVP) signals taken from the MMSE-HR database. The performance of the SB-CWT is compared with the performance of the state-of-the-art Sub-band rPPG (SB).

**Results:**

Median signal-to-noise ratio (SNR) for the proposed SB-CWT ranges from 6.63 to 10.39 dB and for the SB from 4.23 to 6.24 dB. The agreement between the estimated PRs from rPPG pulse signals and the reference signals in terms of the coefficients of determination ranges from 0.81 to 0.91 for SB-CWT and from 0.41 to 0.47 for SB. All the correlation coefficients are statistically significant (*p* < 0.001). The Bland–Altman plots show that mean difference range from 5.37 to 1.82 BPM for SB-CWT and from 22.18 to 18.80 BPM for SB.

**Discussion:**

The results show that the proposed SB-CWT outperforms SB in terms of SNR and the agreement between the estimated PRs from RGB video signals and PRs from the reference BVP signals.

## Introduction

Remote photoplethysmography (rPPG) is a non-contact optical method that measures the intensity of the light reflected from the skin by the means of a digital camera. Formation of the rPPG signal is thought to be attributed to the pulsatile variations of the light absorption due to the pulsatility of the blood volume inside capillaries, which are due to the pumping of the blood by the heart. However, the results of some research (Jonathan & Leahy, 2011; Kamshilin et al., 2011) are not consistent with the aforementioned model and therefore a new model of rPPG signal formation was proposed (Kamshilin et al., 2015). Its concept is as follows (Kamshilin et al., 2015): during the systole the capillary bed is filled with blood. This filling increases the transmural pressure in the capillaries, which results in the compression of the connective tissue and subsequently, the density of the capillary bed is increased. As a result, the amount of the absorbed light increases. In the end-diastole phase the transmural pressure is the lowest and the amount of back-reflected light is maximal. So, in this model the periodic changes of light scattering and absorption due to the deformation of the connective tissue of the skin are presumed to form the rPPG signal (Kamshilin et al., 2015). rPPG signal, just like the conventional contact photoplethysmogram (PPG), consists of the stationary (DC) and varying (AC) part. The former is related to the skin structure and the average blood volume in arterial and venous system (Tamura et al., 2014). The latter reflects the subtle pulsatile changes between systolic and diastolic phase of the cardiac cycle (Tamura et al., 2014). Detection of these minute changes offers assessment of the pulse rate (PR) as well as other physiological parameters: blood pressure (Jeong & Finkelstein, 2016), oxygen blood saturation (SpO_2_) (Verkruysse et al., 2017; Wieringa, Mastik & Van Der Steen, 2005), respiration rate (Zhao et al., 2013), etc.

The important advantage of rPPG in comparison to the conventional contact-based methods, such as electrocardiography (ECG) and PPG is the absence of the physical contact between the measurement device and a subject. This is a highly desirable characteristic, since contact methods impair mobility and can cause bias, whereas patches/cuffs may induce irritation, discomfort, stress and/or pain. In the clinical environment additional disadvantages occur: attached sensors may damage the skin (epidermal stripping), bonding between a parent and a child can be impaired, and sensors/wires can appear as artifacts on medical imaging (Aarts et al., 2013). The aforementioned bias in the contact methods is due to the vasodilation that occurs when external pressure is applied to the skin (Fromy, Abraham & Saumet, 1998). This physiological phenomenon serves as a protective mechanism which prevents pressure-induced ischemia (Fromy, Abraham & Saumet, 1998), but it also changes the physiological state of the human body and consequently influences the measurements. rPPG has therefore huge potential in various medical applications. These include telemedicine, triage, neonatal care, intensive care, operative settings, epilepsy monitoring, sleep studies, mental health monitoring, etc. Besides its use in medicine, rPPG could also be implemented in the driver monitoring systems, affective computing, studies of human-robot and human-computer-robot interaction, sports, surveillance systems, polygraph testing, authentication systems, animal health monitoring, etc.

Remote photoplethysmography can be implemented in two modes, reflectance and transmittance. In the latter, a digital camera and a light source are positioned opposite to each other, with observed tissue in-between, while in the former they are positioned on the same side of the observed tissue. The majority of the proposed rPPG systems operate in reflectance mode and comprise of a digital camera, dedicated lighting and skin. All the challenges arise from these three components and include camera properties, intensity and spectra of light source, skin-tone, skin composition and motion artifacts (Wang, 2017). General framework for the PR extraction from the video recordings consists of five steps: definition of the region-of-interest (ROI) that includes skin pixels, pre-processing of the raw RGB signal extracted from the ROI, extraction of the rPPG signal, post-processing of the extracted rPPG signal and PR estimation.

### Related works

The feasibility of measuring PR by the means of digital camera was first shown on the time-lapse grayscale images (Takano & Ohta, 2007). In search of the most robust algorithm for the extraction of the pulse signal from video recordings, numerous methods have been proposed afterward: color-space-based, blind-source-separation-based (BSS-based), model-based and data-based. Color-space-based extracts the pulse signal from different channels of standard color spaces (Tsouri & Li, 2015; Verkruysse, Svaasand & Nelson, 2008). BSS-based methods can be divided into Independent-Component-Analysis-driven (ICA) (Poh, McDuff & Picard, 2010) and Principal-Component-Analysis-driven (PCA) (Lewandowska et al., 2011) approaches. Model-based methods include chrominance-based (CHROM) (De Haan & Jeanne, 2013), Blood-Volume-Pulse-vector-based (PBV) (De Haan & Van Leest, 2014) and Plane-Orthogonal-to-Skin (POS) (Wang et al., 2017a) algorithms. The last group, data-based methods, includes the Spatial-Subspace-Rotation (2SR) approach (Wang, Stuijk & De Haan, 2015a). Interested readers may refer to thorough reviews of rPPG methods (McDuff et al., 2015; Rouast et al., 2017; Sikdar, Behera & Dogra, 2016), to their algorithmic principles (Wang et al., 2017a) or to the comparison of different steps of PR extraction from the video recordings (Unakafov, 2017).

An important issue regarding the feasibility of the implementation of rPPG in real-world applications is its high sensitivity to different sources of noise. Most of the existing algorithms derive pulse signal from three-dimensional RGB signals and therefore only two distortions can be eliminated by linear combination of all three color channels which is usually not sufficient. This can either be solved by using a camera with multiple bands (McDuff, Gontarek & Picard, 2014) or by algorithmically increasing the dimensionality of the given RGB signals (Wang et al., 2017c). The latter is done by decomposing the raw RGB signals into multiple components by the means of Fourier Transform. The distortion signals are then suppressed in each of the components and the resulting signals are combined into a single one-dimensional rPPG signal. This algorithm is known as Sub-band rPPG (SB) (Wang et al., 2017c).

### Outline of the paper

In this paper, a novel algorithm called Continuous-Wavelet-Transform-based Sub-Band rPPG (SB-CWT) is proposed. SB-CWT uses a different filter bank for the decomposition of the RGB signals than the SB algorithm, namely Continuous Wavelet Transform (CWT) using the analytic generalized Morse wavelet. In CWT, the target signal is correlated with the shifted (translated) and compressed/stretched (also termed dilated or scaled) versions of the analyzing wavelet. By varying the values of the scale and translation parameters the CWT coefficients are obtained. Multiplying these coefficients with their corresponding scaled and translated wavelets provides the constituent wavelets of the analyzed signal. Wavelet transform has the potential to increase the number of the degrees of freedom of the distortion elimination in comparison to the Short Time Fourier Transform (STFT) that was applied in SB. Additionally, wavelet transform provides more accurate information about the time and the frequency of an analyzed signal than STFT. This is achieved by varying the aspect ratio (i.e., ratio between the length of the time window and width of the frequency band) (Ganesan, Das & Venkataraman, 2004). The proposed algorithm is evaluated on the publicly available dataset MMSE-HR (Tulyakov et al., 2016) and compared with the state-of-the-art SB algorithm (Wang et al., 2017c).

The structure of the article is as follows. In the next section, Materials and Methods, the used MSSE-HR dataset is presented, the state-of-the-art SB algorithm used for evaluation of the proposed algorithm is described, the proposed SB-CWT is presented step-by-step, preprocessing steps of the reference signals are shown and the evaluation metrics are defined. In the Results and Discussion sections, the experimental results are presented and discussed. The conclusions are derived in the last section.

## Materials and Methods

### Dataset description

In our research the subset data from the Multimodal Spontaneous Emotion database (Zhang et al., 2016), which includes 2D frontal face video recordings and blood volume pulse (BVP) measurements—MMSE-HR (Tulyakov et al., 2016)—was used. This dataset consists of 102 RGB image sets and corresponding physiological signals of 40 subjects (23 females and 17 males of various ethnic/racial ancestries) performing six different tasks. Table 1 lists all the tasks together with the emotions arousing from them. Image sets (image size: 1,040 × 1,392 pixels) were recorded with RGB 2D color camera (frame rate of 25 fps) of Di3D dynamic imaging system (Dimensional Imaging Ltd, Glasgow, Scotland). BVP signals were measured using Biopac NIBP100D (Biopac System, Inc., Goleta, CA, USA) with the sampling rate of one kHz and collected by Biopac MP150 data acquisition system (Biopac System, Inc., Goleta, CA, USA). All the recorded data were synchronized.

**Table 1:** Performed tasks eliciting various emotions during the video recordings of the subjects in the MMSE-HR database.

Task no.	Task identification	Task description	Target emotion
1	T1	Listen to a funny joke (interview)	Happiness, amusement
2	T8	Improvise a silly song	Embarrassment
3	T9	Follow-up task similar to T8	Embarrassment
4	T10	Experience physical threat in dart game	Fear, nervousness
5	T11	Submerge hand into ice water	Physical pain
6	T14	Experience smelly odour	Disgust

**Note:**

Adopted from Zhang et al. (2016).

### Sub-Band rPPG (SB) method

The proposed SB-CWT method is benchmarked against the SB algorithm (Wang et al., 2017c). This method uses Fourier Transform for transforming the raw RGB signals into the frequency domain. The signals are then projected onto the plane termed as POS (Wang et al., 2017a), the axes of which most likely encapsulate the pulsatile region. The exact projection direction is then localized using the alpha-tuning (De Haan & Jeanne, 2013). Projection to POS together with alpha-tuning serves for the extraction of the rPPG (pulse) signal from the decomposed raw RGB signals. Next, the individual sub-band pulse signals are weighted using the ratio between the pulsatile amplitude and intensity variation amplitude as the weighting function. Finally, Inverse Fourier Transform is used for the transformation of the signal back into the time domain.

### Choosing the analyzing wavelet

**Figure 1:**
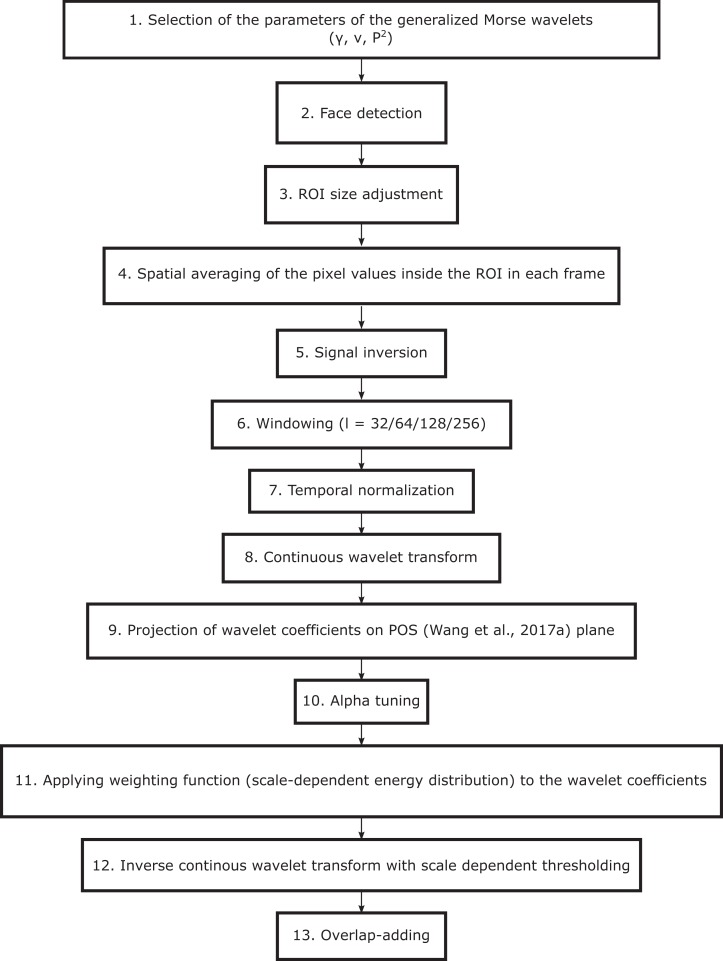
Framework of the proposed SB-CWT method.

**Figure 2:**
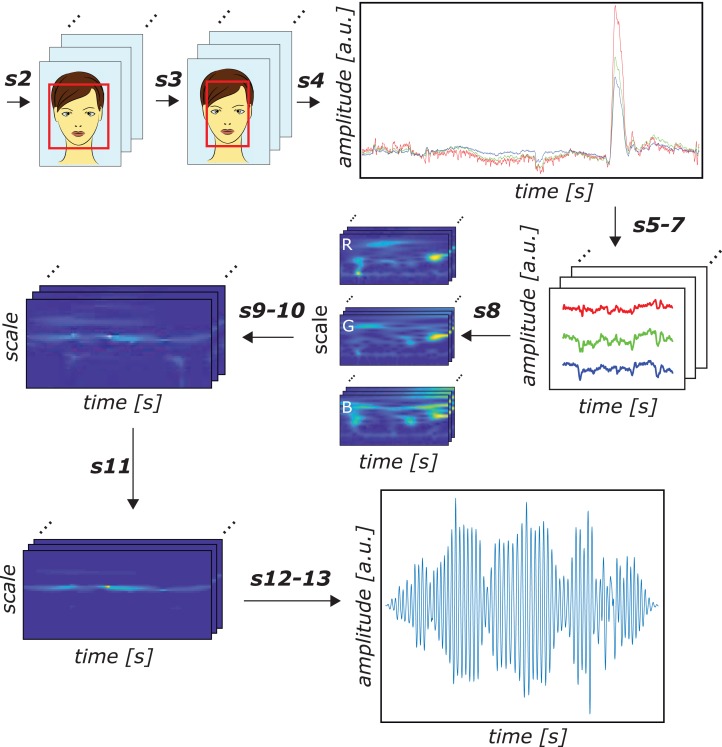
Schematic representation of the proposed SB-CWT method. The illustration shows the entire procedure of extracting the pulse rate signal from facial recordings using the proposed SB-CWT. Notations above the arrows refer to the steps of the proposed algorithm. Notation s2 refers to the face detection, s3 to the ROI size adjustment, s4 to the spatial averaging of the pixel values inside the ROI in each frame, s5–7 to the signal inversion, windowing (*l* = 256) and temporal normalization, s8 to the continuous wavelet transform, s9–10 to the projection of wavelet coefficients on POS plane and alpha tuning, s11 to the application of weighting function to the wavelet coefficients, s12–13 to the inverse continuous wavelet transform with scale dependent thresholding and overlap-adding. The numbering of all the steps closely follows the numbering in the framework of the proposed algorithm depicted in Fig. 1. Notes: a.u. denotes the arbitrary units, R, G and B denote red, green and blue color channels, respectively. Colors of the signals in amplitude vs. time plots correspond to the RGB color channels. The output plot of s4 shows zero-mean raw RGB signals.

The general framework of the proposed SB-CWT algorithm is shown in Fig. 1 and the schematic representation of the algorithm is shown in Fig. 2. The framework consists of 13 steps all of which are explained throughout this section. The first question that emerges in any wavelet-based research is which wavelet is the most appropriate for a given application. In the case of using CWT there are several candidate analytic wavelets, such as Morlet, derivative of Gaussian, Shannon, log Gabor, etc. It was shown that there is a wavelet superfamily called generalized Morse wavelets in which all of the aforementioned wavelets can be subsumed (Lilly & Olhede, 2012). Furthermore, it is even possible to objectively define which wavelet within this family is the most appropriate one to be used (Lilly & Olhede, 2012). General Fourier-domain form of the generalized Morse wavelets is:

(1) $${{\hat{\rm\psi }}_{{\rm{\beta\gamma }}}}\left({\rm{\omega }} \right) = U\left({\rm{\omega }} \right){a_{{\rm{\beta\gamma }}}}{{\rm{\omega }}^{\rm{\beta }}}{e^{-{\rm{\omega \gamma }}}}$$

where *U*(ω) is the unit step function, *a_βγ_* is the normalizing constant, ω denotes frequency, β is the compactness or the order parameter and γ is the symmetry or the family parameter. Increasing β results in the increased number of oscillations in the time domain and consequent narrowing of the wavelet in the frequency domain. Symmetry parameter γ defines the shape of the wavelet in the frequency domain. The Eq. (1) holds for ω > 0 with ${{\hat{\rm \psi }}_{{\rm{\beta }}{\rm{\gamma }}}}\left( {\rm{\omega }} \right) = 0$ elsewhere (due to the unit step function). Generalized Morse wavelets are controlled by two parameters, β and γ. The next step was therefore to adjust their values. Symmetry parameter γ was set to 3, because the family of wavelets with this γ value possesses a few desirable properties, which make it recommended for general use (Lilly & Olhede, 2012). The γ = 3 family is known as Airy family. Interested readers may refer to Lilly & Olhede (2012) for more details. The only parameter left that needed to be defined was β. This parameter can be defined as *P*^2^/γ, where *P*^2^ is the time-bandwidth product. In our example, *P*^2^ value was defined for each of the four window lengths l (32, 64, 128 and 256 frames) in such a way that the value of the minimum wavelet bandpass frequency corresponds to the minimum frequency within the human heart rate band (40, 240) BPM. The β value was determined accordingly. The relation between a scale and a pseudo-frequency ω_*a*_ (this notation is used because the mapping between a wavelet scale to a frequency can be only done in a general sense) is defined as:

(2) $${{\rm{\omega }}_a} = {1 \over {a {\rm{FF}}}}{f_{{\rm{s}}\_{\rm{cam}}}},$$

where ${f_{{\rm{s}}\_{\rm{cam}}}}$ is a camera frame rate, *a* denotes a scale and FF is the Fourier factor. The maximum scale (corresponds to the minimum wavelet bandpass frequency) can be calculated as *N*/2σ_*t*_, where *N* is signal length and σ_*t*_ is time-domain standard deviation of generalized Morse wavelet. The latter can be approximated by $\sqrt {{P^2}/2}$. For the definition of σ_*t*_ refer to Eq. (21) in Lilly & Olhede (2009). Fourier factor is defined as $2{\rm{\pi }}/{\left({{\rm{\beta }}/{\rm{\gamma }}} \right)^{1/{\rm{\gamma }}}}$. For example, given ω_*a*_ = 40 min^−1^ and ${f_{{\rm{s\_cam}}}}\, = 25\,{\rm{fps}}$, the maximum scale that corresponds to the ω_*a*_ equals 6.8224 is reached at *P*^2^ = 11.

The next required step was to define how finely the scales were to be discretized. This step was important in our application, because discretization indirectly defines the number of sub-bands. In CWT the base scale is usually defined as 2^1/*ν*^, where *ν* is the number of voices per octave parameter. The name of this parameter refers to the fact that *ν* intermediate steps (i.e., scales) are needed if the scale is increased by an octave (i.e., if it is doubled). By increasing the value of *ν*, the scales are discretized more finely, which results in more sub-bands and consequently in more degrees of freedom for noise suppression. In the present research *ν* was set to 10, which means that the base scale was set to 2^1/10^. The reason for choosing *ν* = 10 (common values of *ν* are 10, 12, 14, 16 and 32) was that we did not want to increase the computational time. Increasing *ν* namely requires more computations, which results in longer processing time of the algorithm. The described procedure of the selection of the wavelet parameters is depicted by step 1 in Fig. 1.

### Estimation of the pulse rate from video recordings

Viola-Jones face detector (Viola & Jones, 2001) was used to detect faces from the first image in each image set (step 2 in Fig. 1). The width of the box containing the detected face was resized to 60%, while the height was kept unchanged (Poh, McDuff & Picard, 2011) (step 3 in Fig. 1). This was done to decrease the number of non-facial pixels in the ROI. Position of the ROI was kept constant throughout the entire image set. Raw RGB signals were obtained by spatial averaging of the RGB pixel intensity values inside the ROI in each frame (De Haan & Jeanne, 2013; Verkruysse, Svaasand & Nelson, 2008) (step 4 in Fig. 1):

(3) $${C_i} = {1 \over {mn}}\mathop \sum \limits_{j = 1}^m \mathop \sum \limits_{k = 1}^n {I_{{i_{jk}}}},$$

where *C_i_* denotes *i*-th spatial RGB mean color channel signal (also termed raw RGB signal), ${I_{{i_{jk}}}}$ denotes pixel intensity value *I* of a pixel located in *j*-th row and *k*-th column in *i*-th color channel, *i* = 1, 2, 3 and denotes R, G and B channels, respectively. Spatial averaging reduces the camera quantization noise. In the next step, the raw RGB signals were inverted (step 5 in Fig. 1) in order to enable a better comparison with the reference pulse signals derived from the BVP signals. Pulse signals were namely obtained in reflection rPPG mode and therefore the signal reaches its peaks in each end-diastole phase, when the intensity of the diffuse- or body-reflection component is maximal. On the other side, the BVP signals from the MMSE-HR database were acquired using the measurement device based on the Penaz’s principle (Penaz, 1973) or Vascular Unloading Technique. This is an approach where the digital blood flow is held constant by automatic inflation or deflation of the cuff attached to the finger. The applied pressure is directly related to the arterial pressure and is the highest during the ejection phase of the systole. Further processing was not carried out on the full-length raw RGB signals, but on the windowed signals using four different window lengths *l* = 32, *l* = 64, *l* = 128 and *l* = 256 (step 6 in Fig. 1). The windowed signals were temporally normalized (De Haan & Jeanne, 2013) (step 7 in Fig. 1) in order to remove the dependency of the raw RGB signals on the average skin reflection color. This normalization is defined as:

(4) $${C_{{n_{wi}}}}\left(t \right) = {{{C_{wi}}} \over {{\rm{\mu }}\left({{{\rm{C}}_{wi}}} \right)}}-1,$$

where ${C_{{n_{wi}}}}$ is the *i*-th temporally normalized windowed RGB color channel signal, *C_wi_* is the *i*-th windowed spatial RGB mean color channel signal, μ(·) is the averaging operator, and *i* = 1, 2, 3 and denotes R, G and B channels, respectively.

Temporally normalized windowed signals were then decomposed using the CWT (step 8 in Fig. 1). Since all ${C_{{n_{wi}}}}$signals are discrete, and the generalized Morse wavelet is defined in Fourier domain, the discretized version of the CWT expressed as an inverse Fourier transform was used:

(5) $${W_{{a_i}}}\left(b \right) = {1 \over N}\mathop \sum \limits_{k = 0}^{N-1} {\hat C_{{n_{wi}}}}\left(k \right)\hat{{\rm \psi }}_a^*\left(k \right){e^{i2{\rm{\pi }}kb/N}},$$

where *a* and *b* (*b* = 0, 1, 2 … *N*−1) are dilation and translation parameters, respectively, ${\hat C_{{n_{wi}}}}\left(k \right)$ is the discrete Fourier transform of the *i*-th temporally normalized windowed RGB signal, ${\hat{\rm \psi }}_a^*\left(k \right)$ is the complex conjugate of the Fourier-domain form of the wavelet ψ*_a_*, *N* denotes signal length and *k* denotes index of frequency (*k* = 0, 1, 2 … *N−*1). To ensure unit energy at each scale, the following normalization was used:

(6) $${{\hat{\rm \psi }}_a}\left({a{\rm\omega _k}} \right) = \sqrt {{{2{\rm{\pi }}a} \over {{\rm{\Delta }}t}}} {\hat{\rm \psi }}\left({a{{\rm{\omega }}_k}} \right),$$

where ${\rm{\Delta }}t = 1/{f_{{\rm{s\_cam}}}}$ and ${{\rm{\omega }}_k} = 2{\rm{\pi }}k/N{\rm{\Delta }}t.$ Combining Eq. (5) with Eq. (6) the CWT can be expressed as:

(7) $${W_{{a_i}}}\left(b \right) = {1 \over N}\sqrt {{{2{\rm{\pi }}a} \over {{\rm{\Delta }}t}}} \mathop \sum \limits_{k = 0}^{N-1} {\hat C_{{n_{wi}}}}\left({{{2{\rm{\pi }}k} \over {N{\rm{\Delta }}t}}} \right){{\hat{\rm \psi }}^*}\left({a{{2{\rm{\pi }}k} \over {N{\rm{\Delta }}t}}} \right){e^{i2{\rm{\pi }}kb/N}}.$$

The Eq. (7) was repeated for all scales *a* and all values *b* and the final result *W_i_*, where *i* = 1, 2, 3, was a matrix of wavelet coefficients with the number of rows equal to the total number of scales and the number of columns equal to the signal length *N*.

In order to obtain the pulse signal all the wavelet coefficients were then projected on POS plane (Wang et al., 2017a) (step 9 in Fig. 1). POS is based on the physiological reasoning and uses a projection plane that is orthogonal to the temporally normalized skin-tone direction. The POS algorithm was chosen because it offers the best general performance in extracting PR from RGB signals (Wang et al., 2017a). The axes of the POS projection plane (*P_p_*) are defined as:

(8) $${P_p} = \left( {\matrix{ {\matrix{ 0 & 1 & { - 1} \cr { - 2} & 1 & 1 \cr } } \cr \cr } } \right).$$

Projection of the wavelet coefficients on *P_p_* results in two projected components, *S_1_* and *S_2_*:

(9) $$\matrix{ {{S_1} = {W_2} - {W_3}}, \hfill \cr {{S_2} = - 2{W_1} + {W_2} + {W_3}}. \hfill \cr } $$

The components *S*_1_ and *S*_2_ have in-phase pulsatile and anti-phase specular components which can be algorithmically separated (Wang et al., 2017a). This can be done by the means of the so called alpha-tuning (De Haan & Jeanne, 2013) (step 10 in Fig. 1), which can, for the case of the applied POS algorithm, be expressed as (Wang et al., 2017a):

(10) $$h = {S_1} + {\rm{\alpha }}{S_2,}$$

where α = |*S*_1_|/|*S*_2_|, where |·| denotes complex magnitude operator. In the case when the pulsatile variation is stronger than the specular (*S*_1_ and *S*_2_ are in in-phase), the strength of the resulting signal *h* is enhanced (Wang et al., 2017a). In the case when specular variations prevail (*S*_1_ and *S*_2_ are in anti-phase), the α moves the specular variation strength of one signal closer to the other, which results in the specular variation being cancelled out (due to the addition of two signals that are in anti-phase) (Wang et al., 2017a). In the next step, the obtained sub-band pulse signals were weighted using the global (scale-dependent) energy distribution function (step 11 in Fig. 1) defined as the sum of the original wavelet coefficients (the ones obtained by the CWT) at each scale (Bousefsaf, Maaoui & Pruski, 2013). The *i*-th weighted sub-band (i.e., at *i*-th scale) is defined as:

(11) $${P_{{w_i}}} = \mathop \sum \limits_{j = 1}^n \left| {{h_{ij}}} \right| \cdot {h_{i*}},$$

where ${P_{{w_i}}}$ denotes *i*-th weighted sub-band pulse signal, *h_ij_* denotes the CWT coefficient at *i*-th scale and *j*-th translation parameter, h_i*_ denotes all CWT coefficients at *i*-th scale and *n* is the total number of translation parameters. After the weighting, the discretized Inverse Continuous Wavelet Transform (ICWT; step 12 in Fig. 1) together with scale-dependent thresholding was performed in order to transform the weighted sub-band pulse signals back into the time domain. Scale-dependent thresholding allows transformation over selected scales only. The inverses of the selected scales (i.e., frequencies) lie within the range of human heart rate band (i.e., 40–240 BPM). The transformation was performed by summing of the scaled weighted sub-band signals ${P_{w{\rm{\_scaled}}}}$ over the selected scales only:

(12) $$\tilde P = \mathop \sum \limits_{i = {i_{{\rm{min}}}}}^m {P_{w{\rm{\_scale}}{{\rm{d}}_i}}},$$

where *i* runs from *i*_min_ that is equal to the index of the scale that corresponds to the pseudo-frequency 240 BPM to the index of the last scale, which corresponds to the pseudo-frequency 40 BPM. The scaled weighted sub-band signals are defined as:

(13) $${P_{w{\rm{\_scaled}}}} = 2\log {2^{{1 / \nu }}}{1 \over {{C_{\rm{\psi }}}}}{\rm{real}}\left({{P_w}} \right),$$

where real(·) denotes the operator that takes only the real part of the complex input and *C*_ψ_ is the admissibility constant defined as ${a_{{\rm{\beta \gamma }}}}/{{\Gamma }}\left({{\rm{\beta }}/{\rm{\gamma }}} \right)$ (Γ denotes gamma function). This constant ensures that the wavelet of interest has zero mean. Final pulse signal was obtained by overlap adding zero-mean segments of the pulse signal $(\tilde P)$ using a sliding window with a sliding step of a one time sample (De Haan & Jeanne, 2013; Wang et al., 2017a) (step 13 in Fig. 1).

Average PR estimation from the obtained rPPG pulse signals was based on the average length of interbeat intervals (IBIs) of the entire pulse signal. The IBIs are defined as the intervals between the consecutive systolic peaks. These were detected using the derivative-based method for finding local maxima. The minimum peak-to-peak distance was set to 0.25 s, which corresponds to the upper limit of the human heart rate band (i.e., 240 BPM). This was the only constraint applied in the rPPG signal peak detection algorithm. Extraction of the pulse signal using the state-of-the-art SB method closely followed its original algorithm (Wang et al., 2017a). The only difference is that before the average PR estimation, the pulse signal was inverted due to the same reasoning as explained above.

To summarize, in the proposed SB-CWT the raw RGB signals extracted from the facial images were decomposed applying the CWT. Wavelet transform coefficients were then projected to the POS plane, the axes of which most likely encapsulate the pulsatile region (Wang et al., 2017a). Next, alpha-tuning (De Haan & Jeanne, 2013) was used to localize the exact projection direction. After that the projected coefficients were weighted using the global (scale-dependent) energy distribution function and finally inverted using the ICWT. The comparison of the algorithmic steps of SB-CWT and SB is shown in Table 2. From this table it can be seen that the two methods differ in signal decomposition step, weighting of the sub-band signals and in the transformation of the sub-band signals back into the time domain.

**Table 2:** Comparison of the algorithmic steps of SB and the proposed SB-CWT.

	SB (Wang et al., 2017c)	SB-CWT
Signal decomposition	FFT	CWT using generalized Morse wavelet
rPPG signal extraction	POS (Wang et al., 2017a) + alpha tuning (De Haan & Jeanne, 2013)	POS (Wang et al., 2017a) + alpha tuning (De Haan & Jeanne, 2013)
Weighting function	Ratio between the pulsatile amplitude and intensity variation amplitude	Scale-dependent energy distribution
Signal transformation back into the time domain	IFFT	ICWT

**Note:**

CWT, continuous wavelet transform; FFT, fast Fourier transform; ICWT, inverse continuous wavelet transform; IFFT, inverse fast Fourier transform; POS, Plane-Orthogonal-to-Skin; rPPG, remote photoplethysmography.

### Processing of the reference signals

Pre-processing of the reference signals included the following steps: bandpass filtering, clipping and squaring. Applied bandpass filter was zero phase first-order Butterworth filter with a lower cut-off frequency of 0.67 Hz and upper cut-off frequency of two Hz (these frequencies correspond to 40 and 120 BPM). Filtered signals were then clipped in order to keep only the parts of the signals that are above zero. By subsequent squaring of the signals, noise and diastolic waves were suppressed, while the systolic peaks were emphasized. All the pre-processing steps served for a robust detection of systolic peaks, needed for the estimation of the reference average PR. This was done in the same manner as described above for the rPPG signals, except for the minimum peak-to-peak distance between two consecutive peaks being empirically set to 0.3 s.

### Evaluation metrics

In order to evaluate the performance of the proposed algorithm several metrics were used: Signal-to-Noise ratio (SNR), Mean Absolute Error (MAE), Mean Percentage Error (MPE), Root Mean Square Error (RMSE) and coefficient of determination *r*^2^. SNR was defined as the ratio of the energy spectrum within five bins around the PR frequency (defined as the peak frequency in the spectrum of the reference BVP signal) and the remaining energy contained in the spectrum. Due to the fact that pulse signal is pseudo-periodic, SNR was calculated within a time interval defined by the 256-frames-long sliding window and sliding step of one frame. The chosen SNR metric is a modified version of the metric proposed by De Haan & Jeanne (2013). Mathematical expression of the chosen SNR metric is:

(14) $${\rm{SNR}} = 10{\log _{10}}\left({{{\mathop \sum \nolimits_{f = 40}^{240} {{\left({{w_t}\left(f \right)S\left(f \right)} \right)}^2}} \over {\mathop \sum \nolimits_{f = 40}^{240} {{\left({1-{w_t}\left(f \right)S\left(f \right)} \right)}^2}}}} \right)$$

where *w_t_* (*f*) is a binary window, *S* (*f*) is the spectrum of the rPPG signal obtained by the Fourier transform and *f* is the frequency in BPM.

Mean absolute error measures the absolute average errors’ magnitude. Its formula is:

(15) $${\rm{MAE}} = {1 \over n}\mathop \sum \limits_{i = 1}^n \left| {{\rm{P}}{{\rm{R}}_{{\rm{rPP}}{{\rm{G}}_i}}}-{\rm{P}}{{\rm{R}}_{{\rm{re}}{{\rm{f}}_i}}}} \right|,$$

where ${\rm{P}}{{\rm{R}}_{{\rm{rPP}}{{\rm{G}}_i}}}$ denotes the *i*-th average PR value obtained from rPPG signals, ${\rm{P}}{{\rm{R}}_{{\rm{re}}{{\rm{f}}_i}}}$ is the *i*-th reference average PR value and *n* denotes the total number of facial recordings.

Root mean square error is defined as the square root of the average of squared differences between the estimated $({\rm{P}}{{\rm{R}}_{{\rm{rPP}}{{\rm{G}}_i}}})$ and actual values ${\rm{(P}}{{\rm{R}}_{{\rm{re}}{{\rm{f}}_i}}})$:

(16) $${\rm{RMSE}} = \sqrt {{1 \over n}\mathop \sum \limits_{i = 1}^n {{\left({{\rm{P}}{{\rm{R}}_{{\rm{rPP}}{{\rm{G}}_i}}}-{\rm{P}}{{\rm{R}}_{{\rm{re}}{{\rm{f}}_i}}}} \right)}^2}} .$$

Mean percentage error is the average of the percentage error by which the estimated values $({\rm{P}}{{\rm{R}}_{{\rm{rPP}}{{\rm{G}}_i}}})$ differ from the reference values ${\rm{(P}}{{\rm{R}}_{{\rm{re}}{{\rm{f}}_i}}})$:

(17) $${\rm{MPE}} = {{100\% } \over n}\mathop \sum \limits_{i = 1}^n {{{\rm{P}}{{\rm{R}}_{{\rm{re}}{{\rm{f}}_i}}}-{\rm{P}}{{\rm{R}}_{{\rm{rPP}}{{\rm{G}}_i}}}} \over {{\rm{P}}{{\rm{R}}_{{\rm{re}}{{\rm{f}}_i}}}}}.$$

Coefficient of determination *r*^2^ defines the proportion of the variance in the dependent variable *x* predictable from the independent variable *y*. In the case of a linear least square regression, *r*^2^ is equal to the square of the Pearson correlation coefficient *r*, which is a measure of the linear dependence of two variables:

(18) $$r = {{\sum\nolimits_{i = 1}^n {\left( {P{R_{rPP{G_i}}} - \overline {P{R_{rPPG}}} } \right)\left( {P{R_{re{f_i}}} - \overline {P{R_{ref}}} } \right)} } \over {\sqrt {\sum\nolimits_{i = 1}^n {{{\left( {P{R_{rPP{G_i}}} - \overline {P{R_{rPPG}}} } \right)}^2}} } \sqrt {\sum\nolimits_{i = 1}^n {{{\left( {P{R_{re{f_i}}} - \overline {PRref} } \right)}^2}} } }},$$

where $\overline {{\rm{P}}{{\rm{R}}_{{\rm{rPPG}}}}} $ and $\overline {{\rm{P}}{{\rm{R}}_{{\rm{ref}}}}}$ are the mean values of the estimated and reference PR values, respectively.

The agreement between the reference pulse signal and rPPG signals was assessed using the Bland–Altman plots. In the original plot the differences between the results of the two methods are plotted against their averages (Bland & Altman, 1986). We however used the plots in which the differences between the results are plotted against the reference method (Krouwer, 2008).

## Results

We used 101 RGB image sets from the MMSE-HR database in our study. One image set was eliminated (subject F020 performing task T1), because of the corrupt BVP reference signal preventing the successful extraction of the reference pulse signal. The average size of the ROI was 712 ± 44 × 427 ± 27 pixel (expressed as mean ± standard deviation).

**Figure 3:**
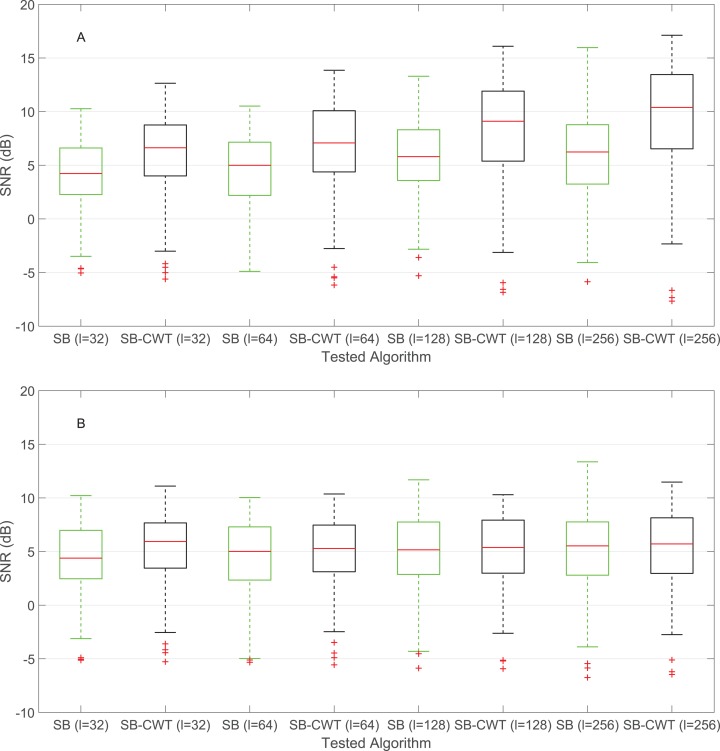
SNR comparison of the SB and the proposed SB-CWT method for four different window lengths (*l*). (A) Results of the original proposed SB-CWT and state-of-the-art SB methods and (B) Results of the modified SB-CWT and SB methods (without the weighting of the sub-band signals). The red lines in each box indicate the median SNR values, bottom and box borders indicate the 25th and 75th percentiles, the whiskers extend to the largest/smallest values not considered as outliers and the red plus signs mark the outliers.

Figure 3 compares the performance of the state-of-the-art SB method and the proposed SB-CWT method for four different window lengths. Figure 3A shows the results of the full original implementation of SB and SB-CWT, whereas Fig. 3B shows the results when weighting (step 11 in Fig. 1) was not applied in neither of the algorithms. The removal of the weighting step was used to study only the effect of the number of sub-bands on the PR estimation. Results in Fig. 3A show that the proposed SB-CWT algorithm outperforms the SB algorithm from the perspective of the median SNR values for all the chosen window lengths. The lowest SNR for both algorithms is achieved at the shortest window length (6.63 dB for SB-CWT and 4.23 dB for SB). The SNR increases with the increasing window length and at *l* = 256 equals 10.39 dB for SB-CWT (6.24 dB for SB). The highest achieved SNR ratio of the SB (at *l* = 256) is exceeded by the SB-CWT at the shortest window length *l* = 32. Results of the paired *t*-test for all window lengths reject the null hypothesis that pairwise difference between SNR data from SB and SB-CWT has a mean value equal to zero (*p* < 0.001). These results indicate that there is a statistically significant difference between the SNR values of SB and SB-CWT. In the case when weighting function was not applied in neither of the two studied algorithms (Fig. 3B), SB-CWT still outperforms SB for all window lengths. The highest SNR for SB-CWT is achieved at *l* = 32 (5.94 dB) and for SB at *l* =256 (5.53 dB). The trend of increasing SNR with increasing window length from Fig. 3A is not present in Fig. 3B for SB-CWT. There is a statistically significant difference between the SNR values of SB and SB-CWT without applied weighting functions at *l* = 32, *l* = 64 and *l* = 128.

**Figure 4:**
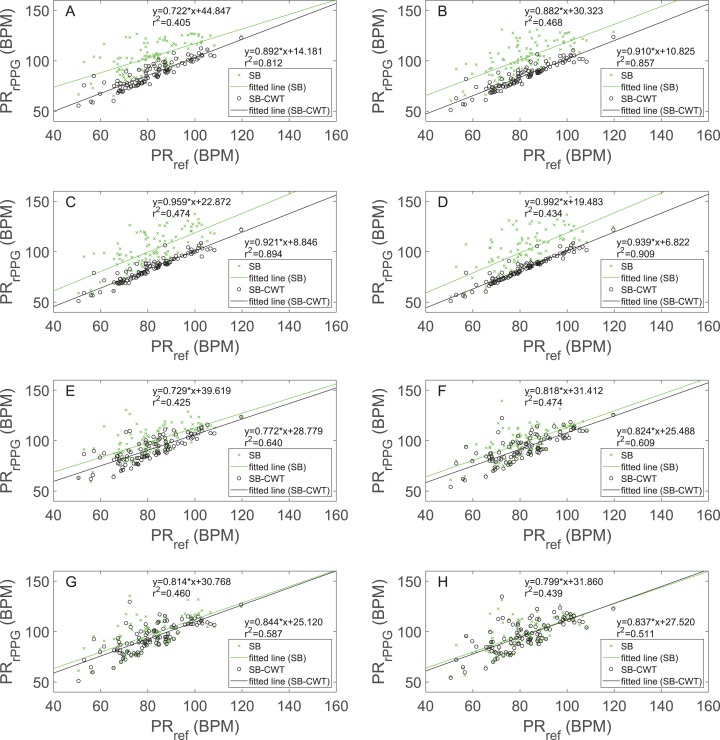
Scatter plots and regression line plots comparing the estimated pulse rates obtained by the proposed SB-CWT and state-of-the-art SB algorithms (PR_rPPG_) with the pulse rates obtained from the reference signal (PR_ref_). Pulse rates estimated from the rPPG signals (PR_rPPG_) are plotted against the pulse rates estimated from the reference pulse signals (PR_ref_) for different window lengths and implementations of the algorithms: (A) Results of the original SB-CWT and SB methods for *l* = 32, (B) Results of the original SB-CWT and SB methods for *l* = 64, (C) Results of the original SB-CWT and SB methods for *l* = 128, (D) Results of the original SB-CWT and SB methods for *l* = 256, (E) Results of the modified SB-CWT and SB methods (without the weighting of the sub-band signals) for *l* = 32, (F) Results of the modified SB-CWT and SB methods (without the weighting of the sub-band signals) for *l* = 64, (G) Results of the modified SB-CWT and SB methods (without the weighting of the sub-band signals) for *l* = 128, (H) Results of the modified SB-CWT and SB methods (without the weighting of the sub-band signals) for *l* = 256. Each subplot shows the equations of the regression lines and coefficients of determination (*r*^2^).

Figure 4 shows the scatter plots examining the relation between the estimated average PRs obtained by both rPPG methods (denoted by PR_rPPG_) and the reference average PRs (PR_ref_) for different window lengths. Linear regression lines together with their equations and goodness of fit (coefficients of determination) are also shown. Figures 4A–4D show the results of the full implementation of both rPPG algorithms, whereas Figs. 4E–4H show the results of both studied algorithms without applied weighting function. Results in Figs. 4A–4D show that with the increasing window length the agreement between the PR_rPPG_ estimated using the SB-CWT and the PR_ref_ is getting higher. In the case of SB the described trend is disrupted at *l* = 256, since the agreement is lower than at *l* = 128. The proposed SB-CWT outperforms SB for all window lengths. Just as it was the case with the SNR, the SB-CWT at the shortest window length outperforms SB at the longest window length. All the correlation coefficients from which coefficients of the determination were calculated are statistically significant (*p* < 0.001). Results in Figs. 4E–4H show that the agreement between the PR_rPPG_ estimated using the SB-CWT and the PR_ref_ is decreasing with the increasing window length. In the case of the SB method, there is no evident trend with increasing window length when it comes to the agreements between PR_rPPG_ and PR_ref_. The range of *r*^2^ values for SB from Figs. 4E–4H are comparable with the values from Figs. 4A–4D.

**Figure 5:**
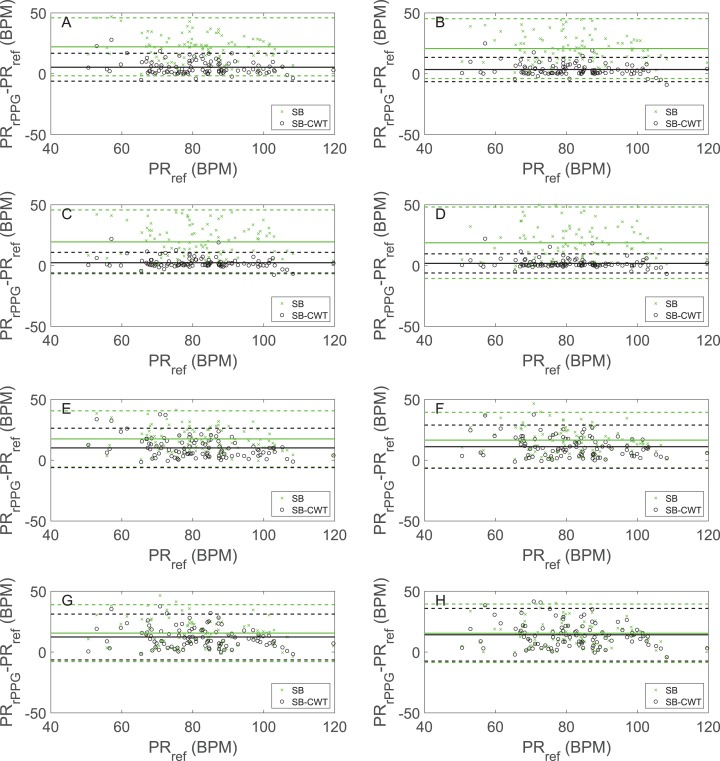
The Bland–Altman plots showing agreements between the estimated pulse rates obtained by the proposed SB-CWT and state-of-the-art SB algorithms (PR_rPPG_) and the pulse rates obtained from the reference signals (PR_ref_). Figure shows the relation between the pulse rates estimated from the rPPG signals (PR_rPPG_) and the reference signals (PR_ref_) for different window lengths and implementations of the algorithms: (A) Results of the original SB-CWT and SB methods for *l* = 32, (B) Results of the original SB-CWT and SB methods for *l* = 64, (C) Results of the original SB-CWT and SB methods for *l* = 128, (D) Results of the original SB-CWT and SB methods for *l* = 256, (E) Results of the modified SB-CWT and SB methods (without the weighting of the sub-band signals) for *l* = 32, (F) Results of the modified SB-CWT and SB methods (without the weighting of the sub-band signals) for *l* = 64, (G) Results of the modified SB-CWT and SB methods (without the weighting of the sub-band signals) for *l* = 128, (H) Results of the modified SB-CWT and SB methods (without the weighting of the sub-band signals) for *l* = 256. The solid lines denote the mean differences. Between the upper and lower limits of the agreement (shown as dashed lines) lie 95% of all the differences.

Figure 5 shows the Bland–Altman plots depicting the agreements between the PR_rPPG_ (obtained by SB and SB-CWT) and the PR_ref_. The subfigures in Fig. 5 are labelled similarly as in Fig. 4. The Bland–Altman plots in Figs. 5A–5D are in agreement with the results in Figs. 4A–4D. The mean differences decrease with the increasing window length. The differences range from 5.37 to 1.82 BPM for SB-CWT and from 22.18 to 18.80 BPM for SB. For the window length *l* = 32, 95% of the measurements fall within −6.11 and 16.85 BPM for SB-CWT (−1.65 and 46.01 BPM for SB). In the case of *l* = 256, 95% of the measurements fall within −6.04 to 9.68 BPM (−10.58 and 48.19 BPM for SB). With the increasing window length, the agreement limits in SB-CWT are becoming narrower. On average, both methods show positive bias on the estimated PRs, but the bias on the proposed SB-CWT is lower than the bias on the SB. The defined intervals of agreement however do not say anything about the acceptability of the SB-CWT. Results in Figs. 5E–5H show increasing mean differences for SB-CWT (ranging from 10.13 to 14.23 BPM) and decreasing mean differences for SB (ranging from 17.52 to 15.47 BPM) with the increasing window lengths. The positive bias is shown on the PR estimation for both methods and for all window lengths. The agreement limits on Figs. 5E–5H are wider than the limits on Figs. 5A–5D.

Statistical comparison of the SB and proposed SB-CWT methods (full implementation of the algorithms) in terms of MAE, MPE and RMSE is shown in Table 3. MAE, MPE and RMSE values decrease with the increasing window length. At *l* = 256, MAE, MPE and RMSE of the estimated PRs using SB-CWT are all more than 7 times lower than MAE, MPE and RMSE of the PRs estimated using SB (at *l* = 256). These results all indicate better performance of SB-CWT when compared to SB.

**Table 3:** Evaluation of the performance of the state-of-the-art SB and the proposed SB-CWT algorithms in terms of MAE, MPE and RMS for four different window lengths *l*.

	MAE (BPM)		MPE (%)		RMSE (BPM)	
	SB (Wang et al., 2017c)	SB-CWT	SB (Wang et al., 2017c)	SB-CWT	SB (Wang et al., 2017c)	SB-CWT
*l* = 32	22.2	5.7	−28.7	−7.1	222.9	54.0
*l* = 64	20.7	4.1	−26.3	−4.6	208.0	35.1
*l* = 128	19.6	3.0	−24.6	−3.2	196.2	23.9
*l* = 256	18.9	2.4	−23.6	−2.5	189.0	18.3

**Note:**

MAE, mean absolute error; MPE, mean percentage error; RMSE, root mean square error.

The computational times of SB-CWT and SB algorithms for the chosen facial recording of the subject F010 performing task 11 for all four window lengths are shown in Table 4. The chosen recording consists of 999 frames, which translates to the total duration of 39.96 s. This video was selected because its duration is equal to the median duration of all the used recordings from the MMSE-HR database. The computational times are expressed as mean ± standard deviation and calculated based on the times of five consecutive executions of the entire code that extracts final pulse signal from the facial recordings. The results show that SB-CWT is computationally more demanding than SB algorithm. All the computations and calculations were performed using the PC with an Intel Core i7 processor and 16 GB of RAM memory. The used software was written in MATLAB R2018a (MathWorks, Inc., Natick, MA, USA).

**Table 4:** Computational times of the state-of-the-art SB and the proposed SB-CWT algorithms for the facial recording of subject F005 performing task 11 (total of 1,552 frames or 62.08 s) for all window lengths.

	*t*_*l* = 32_ (s)	*t*_*l* = 64_ (s)	*t*_*l* = 128_ (s)	*t*_*l* = 256_ (s)
SB (Wang et al., 2017c)	11.25 ± 0.16	11.43 ± 0.30	11.44 ± 0.17	11.32 ± 0.09
SB-CWT	16.51 ± 0.12	17.00 ± 0.19	17.48 ± 0.19	17.41 ± 0.28

**Note:**

Values are expressed as mean ± standard deviation and are calculated based on the computational times of five consecutive executions of the code.

## Discussion

The main reason for comparing the proposed SB-CWT with SB was that SB outperforms POS algorithm (Wang et al., 2017c), whereas POS was reported to have the best overall performance in a large benchmark (Wang et al., 2017a). POS was benchmarked against the single wavelength method (G) (Verkruysse, Svaasand & Nelson, 2008), the method combining two channels (G-R) (Hülsbusch, 2008), PCA (Lewandowska et al., 2011), ICA (Poh, McDuff & Picard, 2011), CHROM (De Haan & Jeanne, 2013), PBV (De Haan & Van Leest, 2014) and 2SR (Wang, Stuijk & De Haan, 2015a) algorithms. All these algorithms were the most commonly used state-of-the-art algorithms at that time.

The proposed SB-CWT algorithm was evaluated on the publicly available MMSE-HR dataset. There are numerous datasets that are in general suitable for the evaluation of the rPPG algorithms (see Table 5), but to the best of our knowledge MMSE-HR is the only one that offers uncompressed facial recordings. This type of recordings is needed for the evaluation of the methods that exploit model-based algorithms for rPPG signal extraction (as is the case with POS algorithm applied in both, SB-CWT and SB). The compression may namely eliminate the pulsating component of the rPPG signal that is related to the blood pulsations and can also pollute the signal with additional artifacts (Wang et al., 2017a). The main reason for publishing the list of datasets that can be used in rPPG studies is to encourage the rPPG community to start using them more commonly. These databases are unfortunately only rarely used by the rPPG researchers, even though most of the proposed algorithms are not based on model-based methods for rPPG signal extraction.

**Table 5:** List of publicly available datasets suitable for rPPG studies.

**DEAP (Koelstra et al., 2012)**
**Participants:** 32 subjects (16 F, 16 M), age: 26.9 (mean)
**Recorded Physiological Data:** BVP, EOG, EMG, EEG, GSR, skin temp., resp. rate
**Video Data:** RGB videos, 720 × 576 @ 50 fps, DV PAL (AVI; h264 codec), duration: 1 min, distance: 1 m, no. of videos: 120
**Illumination Conditions:** controlled illumination (two different indoor environments)
**Citations in rPPG researches:** Qi et al. (2017), Sarkar (2017) and Unakafov (2017)
**MAHNOB-HCI (Soleymani et al., 2012)**
**Participants:** 30 subjects (17 F, 13 M), age: 26.06 ± 4.39 (mean ± st. dev.)
**Recorded Physiological Data:** ECG, EEG, GSR, resp. rate, skin temp., eye gaze data, audio signals
**Video Data:** RGB videos, 750 × 580 @ 61 fps, distance: 0.4 m, duration: various
**Illumination Conditions:** controlled illumination + uncontrolled illumination from the LCD screen
**Citations in rPPG researches:** Hassan et al. (2017a, 2017b), Heusch, Anjos & Marcel (2017), Hsu, Ambikapathi & Chen (2017), Li et al. (2014), Niu et al. (2017) and Tulyakov et al. (2016)
**PURE (Stricker, Müller & Gross, 2014)**
**Participants:** 10 (2 F, 8 M)
**Recorded Physiological Data:** PPG (pulse rate and SpO_2_)
**Video Data:** RGB videos, 640 × 480 @ 30 fps, distance: 1.1 m, duration: 1 min, no. of videos: 60
**Illumination Conditions:** daylight only
**Citations in rPPG researches:** Hsu, Ambikapathi & Chen (2017)
**DECAF (Abadi et al., 2015)**
**Participants:** 30 subjects (14 F, 16 M)
**Recorded Physiological Data:** MEG, EOG, ECG, EMG
**Video Data:** NIR videos, 20 fps
**Illumination Conditions:** controlled illumination
**Citations in rPPG researches:** none
**MMSE-HR (Tulyakov et al., 2016)**
**Participants:** 40 (23 F, 17 M)
**Recorded Physiological Data:** BVP
**Video Data:** RGB image sequences, 1,040 × 1,392 @ 25 fps, duration: various, no. of videos: 102
**Illumination Conditions:** controlled illumination
**Citations in rPPG researches:** Macwan, Benezeth & Mansouri (2018)
**AMIGOS (Miranda-Correa et al., 2017)**
**Participants:** 40 subjects (13 F, 27 M), age: 28.3 (mean)
**Recorded Physiological Data:** audio signals, EEG, GSR, ECG
**Video Data:** RGB videos, 1,280 × 720 @ 25 fps, HD (MOV; h264 codec), recordings of individuals and 4 subjects group
**Illumination Conditions:** controlled illumination
**Citations in rPPG researches:** none
**COHFACE (Heusch, Anjos & Marcel, 2017)**
**Participants:** 40 subjects* (12 F, 28 M), age: 35.60 ± 11.77 (mean ± st. dev.)
**Recorded Physiological Data:** BVP, resp. rate
**Video Data:** RGB videos, 640 × 480 @ 20 fps, MP4 format inside AVI movie container, duration: 1 min, no. of videos: 140
**Illumination Conditions:** (1) controlled illumination only and (2) daylight only
**Citations in rPPG researches:** none
**UBFC-RPPG (Bobbia et al., in press)**
**Participants:** no info
**Recorded Physiological Data:** PPG (pulse rate and SpO_2_)
**Video Data:** RGB videos, 640 × 480 @ 30 fps, distance: 1 m, duration: 2 min, no. of videos: 43
**Illumination Conditions:** no info
**Citations in rPPG researches:** Macwan, Benezeth & Mansouri (2018)
**A dual-mode sleep video database (Hu et al., 2018)**
**Participants:** 12 (2 F, 10 M), age: 21–38
**Recorded Physiological Data:** ECG
**Video Data:** thermal and IR videos, 640 × 480, distance: 1–3 m, no. of videos: 56
**Illumination Conditions:** controlled IR illumination
**Citations in rPPG researches:** none

**Notes:**

BP, blood pressure; BVP, blood volume pulse; ECG, electrocardiography; EEG, electroencephalography; EMG, electromyography; EOG, electrooculography; F, female; GSR, galvanic skin response; IR, infrared; M, male; NIR, near-infrared; PPG, photoplethysmography; resp. rate, respiratory rate; RGB, red, green, blue color space; skin temp., skin temperature; SpO_2_, blood oxygen saturation; st. dev., standard deviation.

* Videos are available for only 22 subjects.

Results from Figs. 3A, 4A–4D, 5A–5D and Table 3 indicate that the proposed SB-CWT outperforms the state-of-the-art SB algorithm. The reasoning behind the better performance is multifaceted. Firstly, at *l* = 32, *l* = 64 and *l* = 128, SB-CWT segments the input rPPG signal on more sub-bands, which in turn increases the effectiveness of the separation of the pulse signal from noise components. SB-CWT (SB) offers 25 (5) sub-bands at *l* = 32, 26 (10) at *l* = 64, 26 (21) at *l* = 128 and 26 (43) at window length *l* = 256. It can be seen that with increasing window length the number of sub-bands in SB is increasing, while it does not change in SB-CWT. The only exception is at *l* = 64, when SB-CWT has one sub-band more than *l* = 32. This is however attributable to the discretization of scale values and their correspondence with the limits of human heart rate frequency band. Secondly, in SB algorithm the weighting function is based on the assumption that noise related to motion causes large intensity variations (Wang et al., 2017c). In the proposed SB-CWT, the weighting function however assumes that the amplitudes of the pulse signals are bigger than those of the noise signals (Bousefsaf, Maaoui & Pruski, 2013). Since the MMSE-HR database does not include very challenging recordings from the perspective of motion artifacts, the selected weighting function in the proposed SB-CWT is more likely more appropriate than the weighting function applied in the SB. This is partially confirmed by the fact that at *l* = 256 the number of sub-bands in SB-CWT is lower than the number in SB, but SB-CWT still performs significantly better.

In order to assess only the effect of the number of sub-bands on the performance of both algorithms, SB-CWT and SB methods were modified in the way that the weighting functions were not applied (step 11 in Fig. 1 was omitted). The results of modified SB and SB-CWT algorithms are shown in Figs. 3B, 4E–4H and 5E–5H. It is interesting that the median SNR value of SB-CWT at *l* = 256 is comparable to that of SB (see Fig. 3B), even though SB has 17 more sub-bands at that window length. It seems that the reason behind this phenomenon is that there are more degrees of freedom than independent distortions. When this is the case, increasing the number of sub-bands does not improve the performance of the algorithm. If weighting functions are applied (Fig. 3A), SB-CWT shows however significantly better performance than SB at *l* = 256. The reasoning behind this fact is that the weighting function applied in SB-CWT assumes that the pulse signal exhibits the strongest amplitude compared to noise signals, which is most likely the case in the studied facial recordings. The results from Figs. 4E–4H and 5E–5H also show that the agreement between the PR_rPPG_ and PR_ref_ does not increase with the increasing window length. This is due to the fact that the applied weighting functions in SB and SB-CWT serve for combining the individual sub-band pulse signals into a single output pulse signal. If there is no weighting applied, the noise-dominated sub-band signals are not suppressed from the individual sub-band pulse signals, which results in a noisier output pulse signal. This is more pronounced at longer window lengths, when the number of frequency bins and consequently the number of sub-band signals is larger. Furthermore, the applied method of estimating average PR from IBIs is very sensitive to noise. This consequently further aggravates the agreement between the PR_rPPG_ and PR_ref_.

Despite the improved performance of SB-CWT in comparison to SB there are some examples, when SB-CWT did not perform better than SB. These examples are shown as outliers in Fig. 3A. These arise from the following subject/task pairs: F013/T8, F018/T1, M007/T10 and M016/T10. There are two main reasons for the poor performance of both: the proposed SB-CWT and the state-of-the-art SB at these examples. Firstly, three out of four subjects listed above are of Hispanic/Latino or African American ethnicities. Their darker skin tone increases the amount of the absorbed light, because of the higher melanin content in the skin. Therefore, the amount of the light that reaches pulsatile blood vessels is reduced which weakens the pulsatile part of the rPPG signal (Wang, Stuijk & De Haan, 2015b). Poorer performance of SB-CWT in the case of darker skin tone is somehow expected, since the algorithm is not designed for addressing the low pulsatility issues, just as it is the case with SB. Secondly, tasks T1 and T8 include more facial and head motion than the other tasks. The motion reduces the amount of the skin pixels inside the ROI with the fixed position and at the same time increases the amount of the non-skin pixels. The effect of poorer facial mask could be however improved by implementing face tracker and/or skin pixel segmentation.

The major advantage of the proposed SB-CWT method is, in general, its ability to offer more sub-bands (by increasing the number of voices per octave) than the SB method, which means that it offers suppression of more noise signals. Next, SB-CWT enables the usage of a weighting function based on the global (scale-dependent) energy distribution, which serves as an additional filter of undesired signal components. Both the advantages result in a more accurate assessment of PR from facial recordings compared to SB. Additionally, an important advantage of SB-CWT can be also seen when real-time applicability of SB-CWT and SB is discussed. SB-CWT namely offers more robust pulse-rate assessment at lower latency when compared to SB. Considering 25 fps camera recordings, the latency at *l* = 32 is 1.28 s, at *l* = 64 2.56 s, at *l* = 128 5.12 s and at *l* = 256 the latency equals 10.24 s. In order to achieve a comparable performance of SB-CWT at *l* = 32, the window length has to be increased to *l* = 256 for SB. In this case, there is an almost 9 s difference between the latencies. Finally, the method was evaluated on publicly available datasets. Therefore, it is easy to reproduce the results and compare them with the results of other algorithms.

On the other hand, SB-CWT has some disadvantages. Firstly, its inherent disadvantage, which is the same as with the SB, is that the algorithm cannot suppress motion that is of the same frequency as the pulse. However, this problem may be solved by applying Amplitude-Selective Filtering (Wang et al., 2017b), which exploits the fact that relative pulsatile amplitude of the rPPG signal varies within a specific range. Secondly, the weighting function based on the global (scale-dependent) energy distribution function assumes that the pulse wave signal exhibits the strongest amplitude compared to the noise signals. This is however not always the case, especially when longer portions of noisy signal are examined. Thirdly, SB-CWT is computationally more demanding than SB. However, the computational times (see Table 4) are still more than two times shorter than the actual duration of the sample facial recording, therefore the processing latency due to the windowing is of greater importance when it comes to assessing the feasibility of SB-CWT in real-world applications. Lastly, the proposed SB-CWT was not tested on the most challenging use case scenarios that involve a lot of motion. This is mostly due to the fact that to the best of our knowledge there is no appropriate publicly available datasets for the motion robustness testing of the proposed SB-CWT. However, it can be assumed that, since SB-CWT offers more sub-bands, it has the potential to offer significant motion robustness. This is however to be studied in the future work.

## Conclusions

The extent of the elimination of the noise signals from the pulse signal in rPPG depends on the dimensionality of the acquired video signal. In order to increase the dimensionality of the RGB signals, SB-CWT algorithm based on the wavelet signal decomposition using generalized Morse wavelet was applied in the present work. The performance of the proposed algorithm was tested on the 101 facial videos from the publicly available dataset MMSE-HR and compared to the state-of-the-art SB algorithm using different window lengths. The results indicate that the proposed SB-CWT algorithm outperforms the state-of-the-art SB algorithm in terms of SNR and agreement between the estimated and the reference PRs. In order to further assess the feasibility of the proposed SB-CWT method in real-world applications, the method needs evaluation on recordings with significant motion.

## Supplemental Information

10.7717/peerj.5859/supp-1 Supplemental Information 1 The MATLAB code used in the research.

## Additional Information and Declarations

### Data Availability

The following information was supplied regarding data availability: Raw data is provided in the Supplemental Files.
